# A Conundrum of Diagnostic Analogy Between Constrictive Pericarditis and Pericardial Tamponade

**DOI:** 10.7759/cureus.9036

**Published:** 2020-07-06

**Authors:** Fateen Ata, Omnia Osman, Saad Javed, Bassam Muthanna, Galal Abushahba

**Affiliations:** 1 Internal Medicine, Hamad Medical Corporation, Doha, QAT; 2 Cardiology, Hamad Medical Corporation, Doha, QAT; 3 Internal Medicine, Allama Iqbal Medical College, Lahore, PAK; 4 Internal Medicine, Hamad General Hospital, Doha, QAT; 5 Cardiology, Our Lady of Lourdes Hospital, Drogheda, IRL

**Keywords:** constrictive pericarditis, pericardial tamponade, echocardiogram

## Abstract

Constrictive pericarditis and cardiac tamponade are two key pathologies of the pericardium. Both increase the intrapericardial pressure and cause adverse effects on the physiological distention and relaxation of the heart’s chambers. They share multiple overlapping features and, therefore, can be very challenging to differentiate between the two with regards to clinical presentation and non-invasive imaging techniques. We present a similar case with a diagnostic challenge from the laboratory investigations and non-invasive imaging. We have discussed the pathophysiology with the common and distinguishing features of the two pathologies when there is an ambiguity.

## Introduction

Constrictive pericarditis (CP) and pericardial tamponade are two different entities with different pathophysiology and treatment modalities. However, infrequently patients can present with comparable clinical features, equivocal electrocardiogram (ECG), and analogous echocardiogram and MRI findings. Subsequently, it may come down to invasive studies, i.e., cardiac catheterization, to differentiate between the two. We present a case in which clinical presentation, basic workup, and advanced non-invasive investigations remained inadequate to distinguish between effusive CP and pericardial tamponade.

## Case presentation

A 23-year-old Indian gentleman presented with a three-week history of shortness of breath on exertion and productive cough with whitish sputum for five days. He also complained of a low-grade fever and pleuritic chest pain for one week. His past medical history was unremarkable. He had a history of alcohol intake and smoking. The patient was vitally stable, other than tachycardia (105 beats per minute). ECG showed sinus tachycardia. A chest X-ray was done, which was grossly unremarkable.

On examination, the patient had a mild decreased intensity of breath sounds over the right infrascapular area and a positive pulsus paradoxus. The rest of the physical examination was unremarkable.

**Figure 1:**
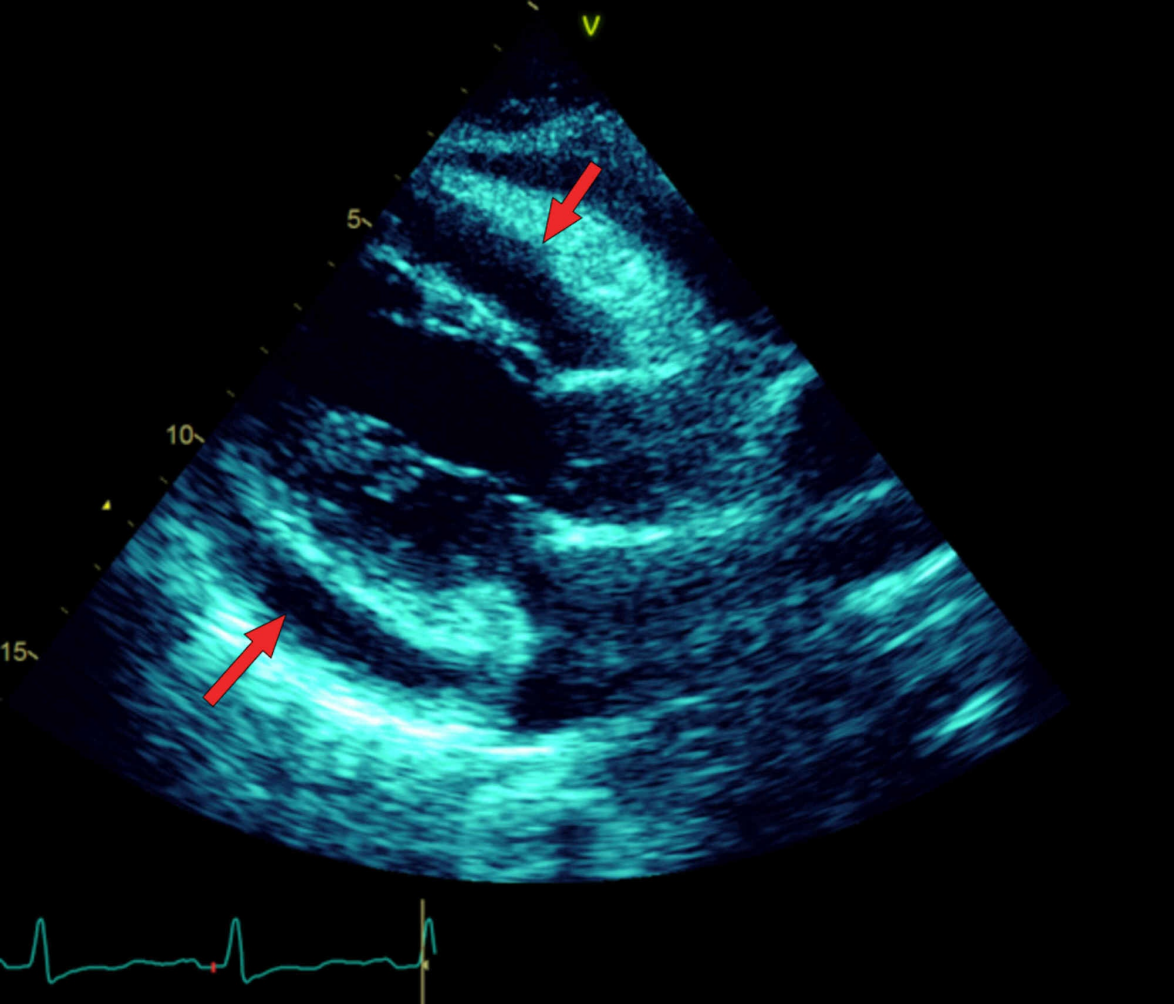
Red arrows: circumferential pericardial effusion (echocardiogram).

A possibility of viral pericarditis with heart failure was considered, and consequently, a transthoracic echocardiogram was ordered, which showed a circumferential pericardial effusion, mainly adjacent to the right ventricle with echo signs of tamponade (Figure 1). The viral panel for common respiratory viruses was negative.

At this point, the diagnosis of pericardial tamponade was considered, and a pericardiocentesis was attempted. Approximately 100 cc of hemorrhagic fluid was aspirated and sent for analysis. The fluid was transudate and did not grow any pathogen.

**Figure 2:**
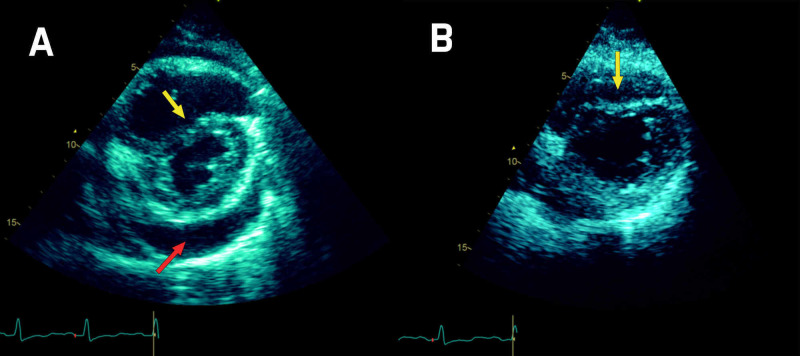
Echo short axis view: pericardial effusion and septal bounce. (A) Pre-drainage: pericardial effusion (red arrow) and septal bounce (yellow arrow). (B) Post-drainage: persistent septal bounce (yellow arrow).

Follow-up echocardiogram revealed a septal bounce (Figure 2) and thickened pericardium. Despite the patient being admitted with pericardial tamponade, minimal fluid could be drained from the pericardium.

**Figure 3:**
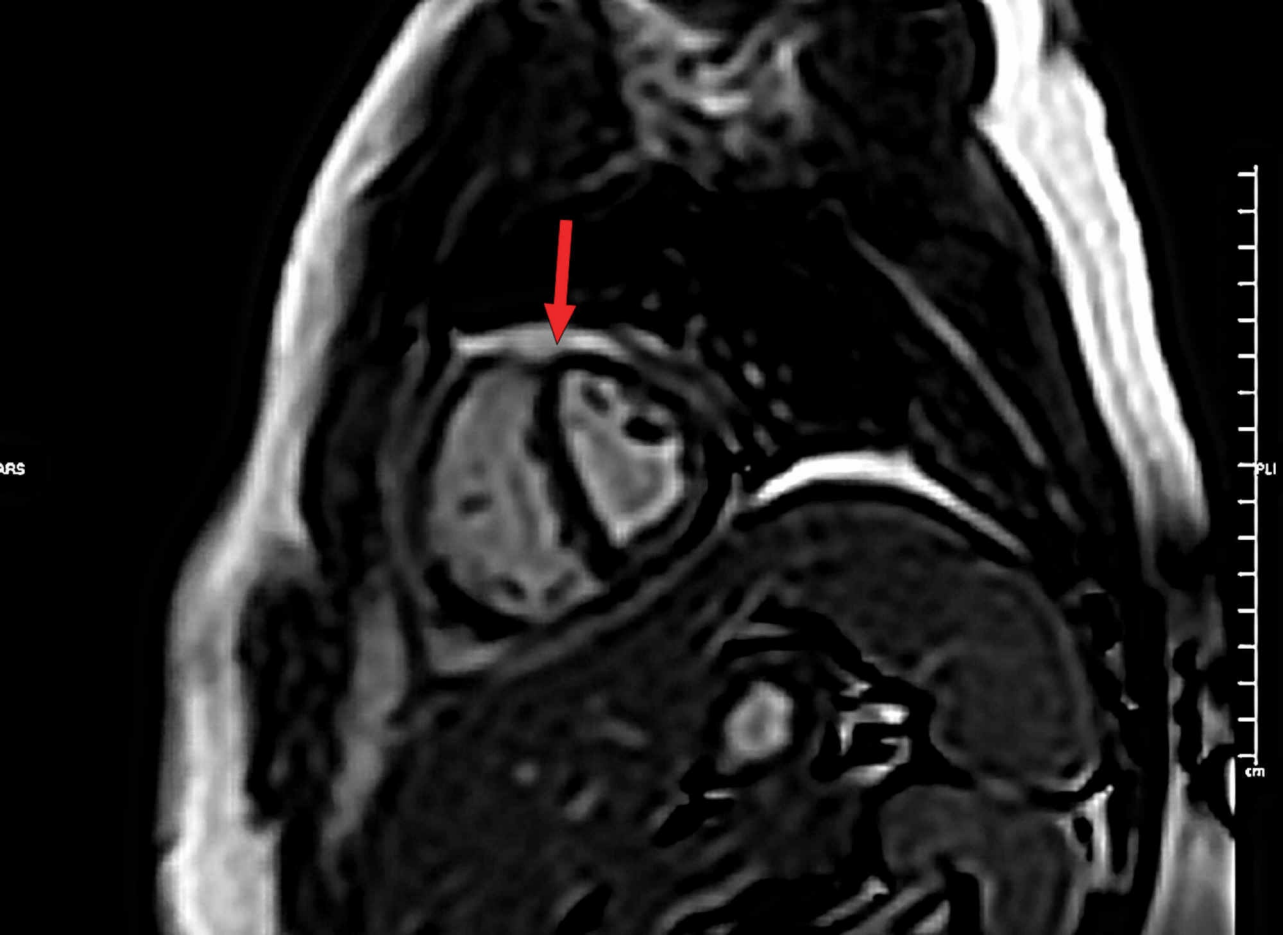
MRI short-axis view. Red arrow: septal bounce.

**Figure 4:**
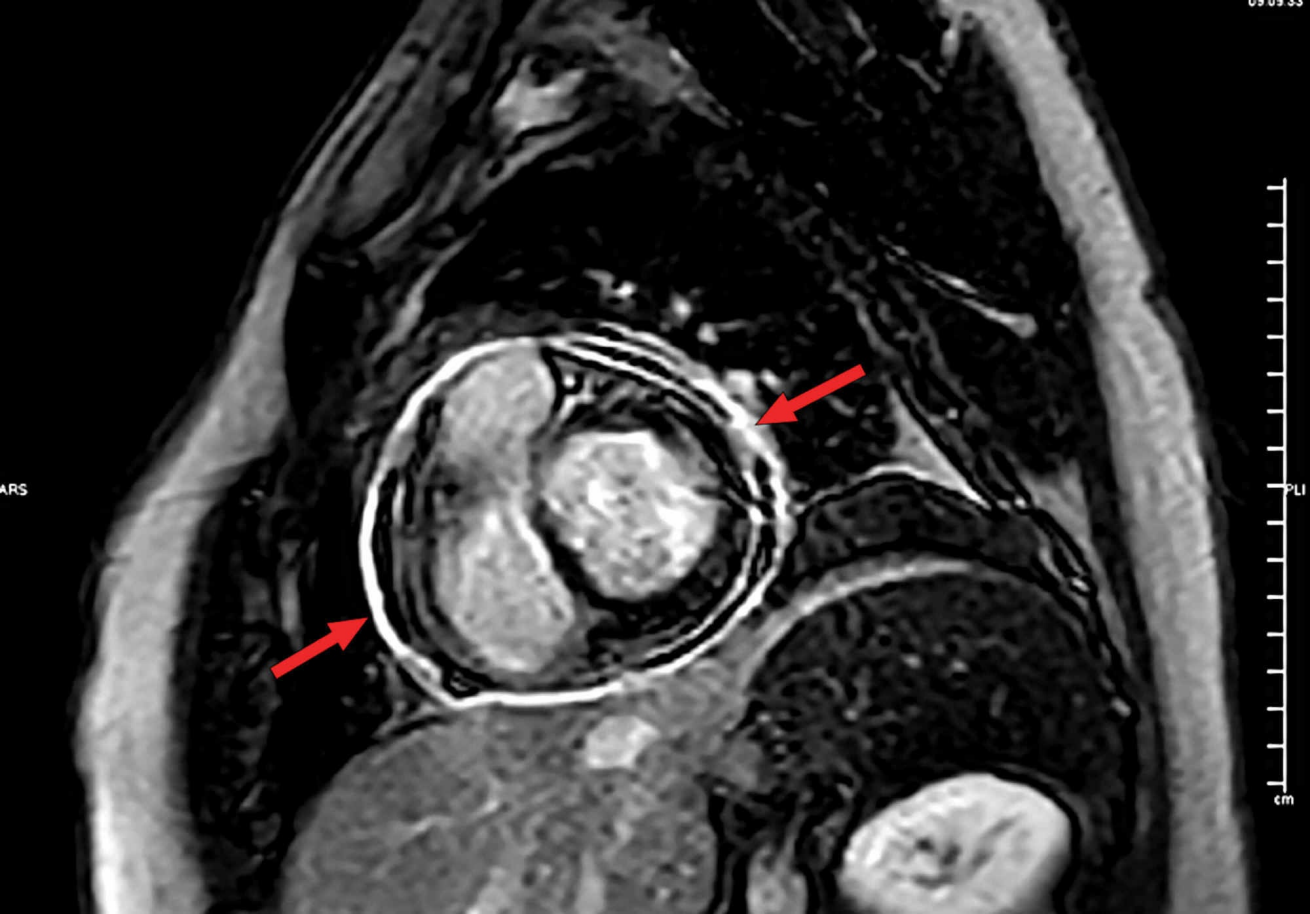
MRI short-axis view. Red arrow: septal bounce (late gadolinium enhancement).

MRI of the heart showed evidence of interventricular septal bounce (Figure 3), and the late gadolinium enhancement images showed pericardial enhancement suggestive of pericarditis (Figure 4). 

Ultimately right heart catheterization was planned to assess the right-sided pressures and confirm the constriction hemodynamics accurately. While admitted, the patient received a course of antibiotics and ibuprofen, and he was discharged in an asymptomatic condition, with a plan of outpatient cardiac catheterization yet to be done.

## Discussion

The pericardium envelopes the cardiac chambers and under physiological conditions exerts subtle functions, including mechanical effects that enhance normal ventricular interactions that contribute to balancing left and right cardiac outputs [1].

Pericardial tamponade is usually an acute or subacute condition in which fluid accumulates between the outer fibrous layer and the inner membranous layer of the pericardium. Because the pericardium is non-compliant, this build-up of fluid causes a rise in the intrapericardial pressure and hence compresses the heart. The common causes include idiopathic, infectious, autoimmune, neoplasms, and trauma.

On the other hand, CP is usually a chronic process of gradual thickening of the pericardium, causing it to lose its elasticity over time. This results in restriction of the heart to expand fully during inspiration to accommodate a venous return, thus causing a decline in pulmonary venous pressure and ultimately reduced left ventricular volume. Common causes include viral, post-radiotherapy, connective tissue disorders, and idiopathic.

CP and pericardial tamponade share many similarities in various aspects. Both can be preceded by viral infections or can be secondary to malignancies or autoimmune diseases. Pulsus paradoxus may also be seen in CP, though with a frequency less than that seen in cardiac tamponade [2]. Other mutual findings may include a raised jugular venous pressure (JVP), sinus tachycardia, and a pericardial rub.

Echo features can be strikingly congruent, for example, ventricular interdependence; the distension of the right ventricle is limited to the interventricular septum, which along with relative underfilling of the left ventricle allows the septal protrusion to the left, decreasing left ventricular compliance and leading to more reduced left ventricle loading during inspiration [3].

MRI is the best suited modality for detecting minor or confined pericardial effusions, pericardial inflammation, and functional irregularities caused by pericardial constriction and pericardial mass characterization. The wide field of view enables assessment of surrounding structures as well [4]. It allows detection of pericardial effusions with high sensitivity, demonstrating fluid collections as small as 30 mL [5]. The ultimate modality to categorically distinguish between CP and cardiac tamponade is invasive, i.e. catheterization, which is also the gold standard for the diagnosis of pericardial constriction [6]

**Figure 5:**
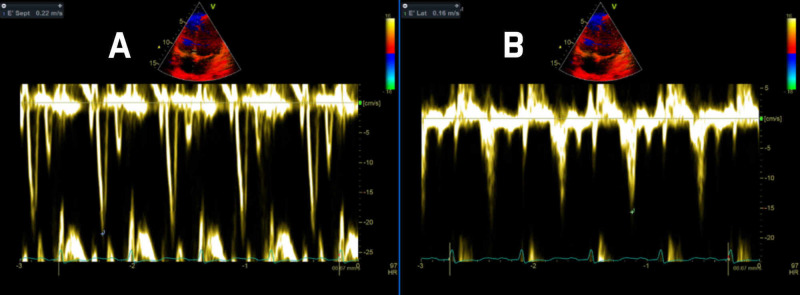
Classic annulus inversus suggestive of the physiology of constrictive pericarditis. (A) Tissue Doppler pulsed wave over septal annulus of the mitral valve. (B) Tissue Doppler pulsed wave over lateral annulus of the mitral valve.

Cardiac tamponade and CP, while having several features in common, do differ in their effect on how they alter ventricular filling. The differences stem from differences in the pattern of restriction to ventricular filling. After the drainage of pericardial fluid, the presence of annulus inversus (Figure 5) (seen in echocardiogram) is highly suggestive of CP. Furthermore, in CP restriction is limited to late diastole, while it is throughout the diastole in cardiac tamponade. This is evident by the rapid 'y' descent, 'dip & plateau' pattern, and pressure equalization during late diastole in cases of CP. On the contrary, in cases involving cardiac tamponade, pressure equalization occurs throughout the diastole. Furthermore, unrestricted thoracic pressure transmission in cardiac tamponade contributes to a retained inspiratory rise in systemic venous return (absence of the Kussmaul sign) and respiratory variability in the right atrial pressure. The preferential inspirational filling of RV, therefore, is secondary to increased filling, rather than reduced left ventricular filling seen in CP [7].

## Conclusions

Although CP and cardiac tamponade are two discrete clinical conditions with respect to etiology, pathophysiology, and management, considerable similarities exist among the two. Sometimes similar clinical features and non-invasive investigations can be insufficient to single out the diagnosis, and eventually cardiac catheterization is required for the diagnostic purpose.
